# COVID-19 Vaccination in Patients With Inflammatory Bowel Disease: Communiqué From the Canadian Association of Gastroenterology

**DOI:** 10.1093/jcag/gwaa046

**Published:** 2021-01-27

**Authors:** Frances Tse, Paul Moayyedi, Kevin A Waschke, Mark MacMillan, Nauzer Forbes, Matthew W Carroll, Nicholas Carman, Grigorios I Leontiadis

**Affiliations:** 1 Division of Gastroenterology, McMaster University, Hamilton, Ontario, Canada; 2 Clinical Affairs Committee, Canadian Association of Gastroenterology; 3 President Elect, Canadian Association of Gastroenterology; 4 President, Canadian Association of Gastroenterology; 5 Division of Gastroenterology and Hepatology, Department of Medicine, McGill University, Montreal, Quebec, Canada; 6 Gastroenterology, Dr. Everett Chalmers Regional Hospital, Dalhousie University, Memorial University, St. John’s, Newfoundland and Labrador, Canada; 7 Division of Gastroenterology and Hepatology, University of Calgary, Calgary, Alberta, Canada; 8 Division of Gastroenterology, Hepatology and Nutrition, Department of Pediatrics, University of Alberta, Edmonton, Alberta, Canada; 9 Division of Gastroenterology, Hepatology and Nutrition, Department of Pediatrics, University of Ottawa, Ottawa, Ontario, Canada

## IMMUNIZATION IN PATIENTS WITH INFLAMMATORY BOWEL DISEASE: WHAT WE KNOW TO DATE

Patients with inflammatory bowel disease (IBD) may be at increased risk of developing certain vaccine-preventable infections such as influenza and pneumococcal pneumonia. The effectiveness and safety of vaccinations may be altered in patients with IBD due to the underlying immune dysregulation inherent to IBD and/or the immunosuppressive therapy that is prescribed for the disease.

The Canadian Association of Gastroenterology (CAG) recently completed a comprehensive and rigorous systematic review and grading of evidence of immunization with inactivated and live vaccines in patients with IBD for a clinical practice guideline.(1, 2) The evidence to date suggests that patients with IBD on immunosuppressive therapy may have a lower immune response to certain vaccines. However, inactivated (or non-live) vaccines are safe with no serious adverse events (SAEs) in patients with IBD regardless of whether or not they are on immunosuppressive therapy. The use of live vaccines in patients with immune-mediated diseases (including patients with IBD) on immunosuppressive therapy is also generally safe, although rare SAEs have been reported (3). Both the US Centers for Disease Control and Prevention (CDC) and the Canadian National Advisory Committee on Immunization (NACI) recommend against *live* vaccines ‘in patients on immunosuppressive therapy equivalent to ≥ 20 mg or 2 mg/kg/day of prednisone for ≥ 14 days’ (4,5).

## RISKS OF CONTRACTING SEVERE ACUTE RESPIRATORY SYNDROME CORONAVIRUS 2 (SARS-CoV-2) INFECTION OR DEVELOPING SEVERE COVID-19 (CORONAVIRUS DISEASE 2019) OUTCOMES IN PATIENTS WITH IBD: WHAT WE KNOW TO DATE

It is unknown whether patients with IBD have a different baseline risk of contracting SARS-CoV-2 infection than people without IBD with similar levels of viral exposure, but it is unlikely that the risk is any lower. The evidence to date suggests that patients with IBD who have COVID 19 (including those on long-term biologics or nonsteroid immunomodulatory therapies) may not have an increased risk of severe outcomes (hospitalization or death) compared to COVID-19 patients without IBD (6). However, recent corticosteroid use may be associated with a higher risk of severe COVID-19 outcomes (6,7). The risk factors for severe COVID-19 outcomes in patients with IBD appear to be similar to those widely recognized in COVID-19 patients without IBD. These risk factors include advanced age and multiple comorbidities (6,7).

## SAFETY AND EFFECTIVENESS OF MESSENGER RNA (mRNA) COVID-19 VACCINES: WHAT WE KNOW TO DATE

Pfizer-BioNTech COVID-19 vaccine (BNT162b2) is a lipid nanoparticle-formulated nucleoside-modified mRNA vaccine that encodes a modified form of the spike protein of SARS-CoV-2. The CDC has systematically evaluated the certainty of evidence for the benefits and harms of Pfizer-BioNTech COVID-19 vaccine from one Phase I randomized controlled trial (8) and one Phase II/III randomized controlled trial (9) using the GRADE approach to inform its recommendations (10,11). A lower risk of symptomatic COVID-19 was observed with vaccination compared to placebo (relative risk [RR] 0.05; 95% confidence interval [CI] 0.02 to 0.10, and absolute risk [AR] 9 fewer per 1000; 95% CI from 9 fewer to 8 fewer). This corresponds to a vaccine efficacy of 95.0% (95% credible interval, 90.3% to 97.6%). The certainty of evidence for symptomatic COVID-19 was rated as high, although there were some concerns for indirectness due to the short duration of follow-up (2 months) (Indirectness: Direct evidence comes from research that directly compares the interventions in which we are interested when applied to the populations in which we are interested and measures the outcomes important to patients. As per the GRADE approach, we can have concerns about indirectness when the population, intervention, outcomes, or comparisons differ from those in which we are interested.). The vaccine was also associated with numerically fewer hospitalizations due to COVID-19 (RR 0.0; 95% CI 0.0 to 1.10) and all-cause deaths (RR 0.50, 95% CI 0.09 to 2.73). The certainty of evidence regarding hospitalization due to COVID-19 and death was downgraded to low and very low respectively due to indirectness and imprecision. In terms of harms, the incidence of SAEs was low and was comparable between the vaccine and placebo arms (incidence rate 0.6% versus 0.5%; RR 1.14; 95% CI 0.89 to 1.47). The certainty of evidence for harms was downgraded to moderate due to indirectness related to the short duration of follow-up and imprecision related to the large sample size generally needed to confidently assess the incidence of rare SAEs. From available data, the vaccine’s efficacy was consistent across genders, age groups, ethnic and racial groups, and people with pre-existing medical conditions. Overall, the efficacy and safety data support a positive balance between benefits and harms for Pfizer-BioNTech COVID-19 vaccine.

The CDC has also systematically evaluated the certainty of evidence for benefits and harms of Moderna COVID-19 vaccine using the GRADE approach (12,13). Data were reviewed from one Phase II randomized controlled trial and one Phase III randomized controlled trial provided to the CDC by the sponsor and the FDA. Similar to Pfizer-BioNTech COVID-19 vaccine, the certainty of evidence for prevention of symptomatic COVID-19 was rated as high (RR 0.06; 95% CI 0.03 to 0.11 and AR 13 fewer per 1000; 95% CI from 13 fewer to 12 fewer) for Moderna COVID-19 vaccine. The certainty of evidence for harms in terms of SAEs was rated as moderate (incidence rate 1.0% versus 1.0%; RR 0.96; 95% CI 0.77 to 1.20). Overall, the efficacy and safety data also support a positive balance between benefits and harms for Moderna COVID-19 vaccine.

It is important to note that specific high-risk or vulnerable populations such as young children, very elderly persons (>85 years of age), pregnant or lactating women, and those who are immunocompromised (either due to immunosuppressive therapy or an immunocompromising condition) were excluded from these randomized clinical trials. Several long-term pharmacoepidemiology studies are to be conducted to assess for rare SAEs in diverse populations including these important subgroups not yet studied.

## CAG ENDORSES THE CDC RECOMMENDATIONS FOR MRNA COVID-19 VACCINES

On December 11, 2020, the Food and Drug Administration (FDA) issued an Emergency Use Authorization (EUA) for Pfizer-BioNTech COVID-19 vaccine in persons aged 16 years and older for prevention of COVID-19. This was immediately followed by an interim recommendation for the use of this vaccine by the CDC’s Advisory Committee on Immunization Practices (ACIP). On December 18, 2020, the FDA issued an EUA for Moderna COVID-19 vaccine in persons aged 18 years and older. This was also followed by an interim recommendation for the use of this vaccine by the CDC’s ACIP.

The CDC (14) also issued guidance for vaccination of special populations including persons with underlying medical conditions and immunocompromised persons:

### Persons With Underlying Medical Conditions

“COVID-19 vaccine may be administered to persons with underlying medical conditions who have no contraindications to vaccination. Phase 2/3 clinical trials demonstrated similar safety and efficacy profiles in persons with some underlying medical conditions, including those that place them at increased risk for severe COVID-19, compared to persons without comorbidities”.

### Immunocompromised Persons

“Persons with HIV infection, other immunocompromising conditions, or who take immunosuppressive medications or therapies might be at increased risk for severe COVID-19. Data are not currently available to establish vaccine safety and efficacy in these groups. Persons with stable HIV infection were included in phase 2/3 clinical trials, though data specific to this group are not yet available. Immunocompromised individuals may still receive COVID-19 vaccination if they have no contraindications to vaccination. However, they should be counseled about the unknown vaccine safety profile and effectiveness in immunocompromised populations, as well as the potential for reduced immune responses and the need to continue to follow all current guidance to protect themselves against COVID-19”.

On the other hand, NACI in Canada has advised that “COVID-19 vaccine **should not** be offered to the following populations excluded from clinical trials until further evidence is available. However, if a risk assessment deems that the benefits of vaccine outweigh the potential risks for the individual (e.g., where the risk of severe outcomes of COVID-19 and risk of exposure to SARS-CoV-2 is high) or for the fetus/infant (in the case of pregnancy/breastfeeding) and if informed consent includes discussion about the insufficient evidence in this population, then a complete series of authorized COVID-19 vaccine **may be** offered to individuals in the following populations: immunosuppressed due to disease or treatment, individuals with an autoimmune condition, pregnant or breastfeeding, and adolescents 12 to 15 years of age (only Pfizer-BioNTech COVID 19 vaccine may be offered)” (15)

The CAG endorses the CDC recommendations for the use of mRNA COVID-19 vaccines in special populations, after assessing the evidence using the GRADE approach. Specifically, in patients with IBD not on immunosuppressive therapy, we *recommend* the COVID-19 vaccine be given (strong recommendation, moderate certainty of evidence). In patients with IBD on immunosuppressive therapy, we *suggest* the COVID-19 vaccine be given (conditional recommendation, low certainty of evidence).

As per the GRADE approach, a strong recommendation means that we are confident that the benefits of following the recommendation clearly outweigh the harms, so the course of action should apply to most patients. A conditional recommendation means that the desirable effects of adherence to a recommendation probably outweigh the undesirable effects, but we are not confident about these tradeoffs due to low or very low certainty of evidence, uncertainty regarding the balance of benefits and harms, uncertainty in patients’ values and preferences or questionable cost-effectiveness. Thus, conditional recommendations require shared decision-making as different choices will be appropriate for different patients. Patients with IBD are not considered immunosuppressed at diagnosis, but subsequently may become immunosuppressed due to IBD therapies. It is important to note that there is no standard definition of immunosuppression. The degree to which immunosuppressive therapy causes clinically relevant immunosuppression is dose related and varies by medication. The medications and doses used in patients with IBD would generally cause less severe immunosuppression compared to patients with active HIV infection or post-transplant treatment.

The certainty of evidence for CAG’s recommendations was based on the CDC’s assessments of the evidence for the general population. In patients with IBD not on immunosuppressive therapy, the certainty of evidence for benefits or harms was not further downgraded compared to the assessment for the general population. Given the high certainty of evidence for benefits and moderate certainty of evidence for lack of harms in patients with IBD not on immunosuppressive therapy, the overall certainty of evidence for CAG’s recommendation for this population is moderate.

However, in patients with IBD on immunosuppressive therapy, the certainty of evidence for both benefits and lack of harms was further downgraded to low due to indirectness of the population. Therefore, the overall certainty of evidence for CAG recommendation for this population is low.

The mRNA technology used in Pfizer-BioNTech and Moderna COVID-19 vaccines has been studied for more than a decade. mRNA vaccines do not contain a live virus and do not carry a risk of causing the viral disease in the vaccinated person. Based on cumulative evidence of vaccinations in patients with IBD, it is biologically implausible for mRNA vaccines to cause catastrophic harms in patients with IBD on or not on immunosuppressive therapy, while other serious harms in these patients are highly unlikely. Based on the evidence of other non-live vaccines administered to IBD patients on immunosuppressive therapy, the efficacy of the COVID-19 vaccine may be similar or slightly reduced, when compared to persons without IBD or patients with IBD not on immunosuppressive therapy, but it is extremely unlikely that the vaccine would be entirely ineffective.

The ongoing SARS-CoV-2 pandemic is having significant impact on public health, and there is no broadly effective treatment currently available. Stopping and/or slowing the pandemic will require a substantial proportion of the population to acquire immunity in order to break the chain of transmission. Hence, a safe and effective vaccine can have a significant impact on the trajectory of the pandemic at this critical time. During a serious pandemic, it is important that we do not deny susceptible subpopulations a vaccine with proven efficacy and safety in the general population, merely because these subpopulations were not studied in the initial clinical trials. Such exclusions from the vaccination program risk harming not only the excluded subpopulations, but also the general population by compromising or delaying the potential for herd immunity. Specifically, for patients with IBD, the GRADE approach allows incorporation of indirect evidence from decades of research on vaccines, and justifies inclusion of these patients in a vaccination program. If new evidence emerges that necessitates revising these recommendations, the CAG will provide timely guidance via its website and its official journal, the Journal of the Canadian Association of Gastroenterology.

## CANADIAN ASSOCIATION OF GASTROENTEROLOGY COMMUNIQUÉ

This communiqué on COVID-19 vaccination in patients with inflammatory bowel disease was developed under the direction of Drs. Frances Tse (CAG Chair Practice Affairs) and Grigorios Leontiadis (CAG VP Clinical Affairs), in accordance with the policies and procedures of the Canadian Association of Gastroenterology (CAG) and under the direction of CAG Clinical Affairs. The communiqué was developed following a thorough consideration of medical literature and the best available evidence and clinical experience. The communiqué aims to provide a reasonable and practical approach to care for specialists and allied health professionals charged with the duty of providing optimal care to patients and families, and can be subject to change as scientific knowledge and technology advance and as practice patterns evolve. The communiqué is not intended to be a substitute for physicians using their individual judgment in managing clinical care in consultation with the patient, with appropriate regard to all the individual circumstances of the patient, diagnostic and treatment options available, and available resources.
